# Recordkeeping, logistics, and translation: a study of homeless services systems as infrastructure

**DOI:** 10.1007/s10502-023-09410-0

**Published:** 2023-02-03

**Authors:** Pelle Tracey, Patricia Garcia, Ricardo Punzalan

**Affiliations:** University of Michigan School of Information, Ann Arbor, MI USA

**Keywords:** Recordkeeping, Infrastructure, Homelessness, Bureaucracy, Care

## Abstract

Homeless services systems provide unhoused individuals access to emergency shelter, subsidized housing, and other life-sustaining resources. In this paper, we present a qualitative study that draws on the experiences of fifteen social service workers to examine how recordkeeping practices sustain homeless services systems and unite a tangled web of institutions and actors, including public housing systems, nonprofit agencies, and local governments. We address the following research questions: How is the infrastructure of homeless services sustained by recordkeeping? How are social service workers affected by increasing recordkeeping demands? In what ways do social service workers work against or ‘find the play’ in this system? To address these questions, we collected interviews and conducted artifact walkthroughs with our study participants. We analyzed the data using an infrastructural lens and found that current recordkeeping practices within homeless services systems comprise an "infrastructure of last resort" that functions logistically, prioritizing efficiency and speed. We also found that social service workers “speak back” to logistification by making the homeless services infrastructure more legible to their unhoused clients through mediation and acts of translation that help to produce better resource outcomes. Our findings show how structuring recordkeeping in ways that privilege efficiency and speed disrupts social service work and interferes with social service workers’ ability to provide care for vulnerable individuals facing life-altering and life-threatening hardships.

## Introduction

People experiencing homelessness and housing insecurity face hardships that cause them to seek support from homeless services systems. Homeless services systems provide emergency shelter, subsidized housing, and a plethora of other resources to some of the most vulnerable people in the USA—those who have no place to live and nowhere else to go. However, receiving support through these systems is often difficult because they are organized as tangled sociotechnical webs consisting of remnants of the welfare state, public housing system, nonprofit agencies, local governments, and capital (Lyon-Callo 2004; Willse 2015).

Recordkeeping is vital to homeless services systems as it unites disparate institutions and actors via a vast constellation of practices, standards, and technologies spanning digital and analog formats. In short, records and recordkeeping practices act as the relational ‘glue’ that holds together the sites of the homeless services system, facilitating the movement of unhoused people through physical and digital space. Although records and recordkeeping are vital to the operation of homeless services systems, very little research has examined these systems as *systems*, that is, as sociotechnical configurations within a larger infrastructure. In this paper, we present a qualitative study that draws on the concept of infrastructure and the experiences of fifteen social service workers to examine how recordkeeping underpins homeless services systems. We specifically address the following research questions: How is the infrastructure of homeless services sustained by recordkeeping? How are social service workers affected by increasing recordkeeping demands? In what ways do social service workers work against or ‘find the play’ in this system?

An infrastructural lens provides a conceptual grounding for addressing these questions and examining recordkeeping as a sociotechnical ‘system of systems’ that is embedded and operates in the background (Star and Ruhleder 1996). Infrastructure also offers an accompanying methodological attunement to instances of breakdown, failure, and maintenance that supported the analysis of the social service workers’ experiences and their recordkeeping practices (Larkin 2013; Star 1999). Using this lens, we argue‌ that current recordkeeping practices within homeless services systems sustain an "infrastructure of last resort" that values logistification and privileges efficiency and speed. We use the phrase "infrastructure of last resort" to signal the importance of examining the impact recordkeeping systems have on vulnerable individuals who are facing life altering and sometimes life threatening, hardships.

Our findings present the experiences and narratives of social service workers as evidence illustrating how a particular way of structuring recordkeeping, which we call logistification, disrupts social service work and interferes with social service workers’ desire to be deeply invested in caring for others. Social service workers “speak back” to logistification by working to make the infrastructure legible to their unhoused clients through revealing some aspect of its inner workings, or by mediating their clients’ narration of their life experiences in order to achieve better outcomes. Our findings also reveal the unintended consequences resulting from increasing volumes of paperwork in logistified contexts and how the drive towards producing more organizational records may change the nature of work in ways that do not benefit the populations practitioners aim to serve.

Over the last thirty-odd years, many archival studies scholars have investigated the sociotechnical nature of records (e.g., Hedstrom 1991; Trace 2002; Yakel 2001). Our study expands this line of inquiry by demonstrating how the concept of infrastructure can provide a framework for understanding why and how recordkeeping has such profound effects on the social world. Infrastructure can elucidate how recordkeeping practices, standards, and technologies impact life in real time, not just after the fact. In our study, an infrastructural lens provided insights into the function of records within homeless services systems and the work practices of social service workers. Importantly, these insights have implications for how we understand the consequences of recordkeeping systems and practices on the lives of marginalized and precarious people.

## Background

### Recordkeeping as a sociotechnical process

Prior research in archival science has shown how bureaucratic and organizational records, such as standardized forms and database entries, can have powerful impacts on human life. Ciaran Trace’s investigation of law enforcement records and Elizabeth Yakel’s study of hospital radiologists provide two paradigmatic examples. The former found that records “cannot be viewed simply as transparent reflections of organizational routines and decision-making processes;” (Trace 2002, p.151) while the latter demonstrated how records were both process and product in the negotiation of “multiple accountabilities”—a deeply social phenomenon rather than a merely technical or procedural one (Yakel 2001, p.233). As these studies demonstrate, archival scholars sought to dig deeper, investigating both the technical and social facets of recordkeeping, or as Trace puts it, records’ “use and purpose” (2002, 153). While Trace notes that use and purpose are not exclusive categories, the distinction is important for highlighting how recordkeeping practices should not be taken for granted by archival scholars as either uncomplicated technical reflections of truth or black boxed social processes.

By challenging the view of recordkeeping as a transparent organizational process, archival studies scholars developed analyses of recordkeeping that foregrounded sociotechnical entanglements and forged new interdisciplinary connections to work in sociology, anthropology, organizational studies, and science and technology studies (STS). As Margaret Hedstrom argued, “Electronic records issues cannot be addressed exclusively through research on technical problems because important social and economic factors shape the decisions that individuals and organizations make about information technology” (Hedstrom 1991, p. 341). Together, the work of these scholars troubled any simplified view of records as straightforward representations of human activity and lived experience.

Concurrent with this focus on sociotechnical frameworks was a new interest in highlighting how power operates vis-a-vis archiving. Work by scholars including Joan Schwartz and Terry Cook (2002) and Verne Harris (2002) represents the beginnings of a sustained critique of what Schwartz and Cook (2002) describe as “the on-going denial by archivists of their power over memory, the failure to explore the many factors that profoundly affect records before they come to the archives, and the continued assumptions by many users of archives that the records presented to them are not problematic” (2). In articulating that “the archival record is at once expression and instrument of power,” Harris (2002, p. 85) opened new avenues for thinkers interested in deeply examining how record production and preservation were not only social in nature, but also had deep social and political impacts. For example, Sexton and Sen (2018) demonstrated that the importance of reflecting and taking actions to mitigate power imbalances when developing a participatory archive of mental health recovery. Their work showed the importance of mitigating power imbalances both in the content and process of developing an archive that contested limited notions of mental health recovery primarily stemming from bureaucratic forms of recordkeeping such as case notes. Other work that examines how archives are both expressions and instruments of power includes the efforts to establish critical archival studies (e.g., Punzalan & Caswell 2016) and “rights in records” (Gilliland & McKemmish 2015) as research programs.

The dual view of recordkeeping as both sociotechnical and imbued with power is an analytic framework that is particularly useful for understanding homeless services systems, and for situating the records concept with regard to infrastructure. It helps tease apart the multiple, overlapping functions that a single record might perform. For example, a case note might simultaneously perform the technical functions of documenting that a case manager met with a client as part of their professional obligations and of listing future tasks that other team members may take up later, while also performing a myriad of social functions such as the discursive construction of the client as sick or deviant or the transference of responsibility for homelessness to the individual. Therefore, we draw on these works because they demonstrated that archival scholars not only *could* investigate the sociotechnical nature of recordkeeping, but also were able to generate unique insight given the field’s deep understanding of the social and political power of records. We build upon this view of recordkeeping as a sociotechnical process imbued with power and hope to contribute scholarship extending the analytic frameworks of the discipline beyond the strict boundaries of archival institutions.

### Recordkeeping as an instrument of state power

In recognizing the powerful impacts of recordkeeping on human life, prior work has called for archival scholars and practitioners to pluralize recordkeeping in ways that “enable records to be reviewed, accessed and analyzed beyond an organization or individual life, for multiple external accountability and memory purposes in and through time and space” (McKemmish et al. 2010, p. 4451). The importance of recordkeeping for supporting accountability and memory is particularly important within bureaucratic contexts where records can be used to exercise state power over vulnerable individuals. Although this piece is, to our knowledge, the first to analyze homeless services systems from the perspectives of archival science, some parallel investigations into bureaucratic contexts exist in the literature. Patricia Garcia’s examination of intersecting immigration and labor classification systems in the case of seasonal agricultural workers and Jennifer Hale Eagle’s analysis of proposed destruction schedules for records of migrant detention provide two examples (Garcia 2014; Eagle 2019; see also Bruner 2019). Both demonstrate how the production and utilization of records in classification is not strictly a unidirectional exercise of State power but also a sort of discursive negotiation between migrants, their employers, and various State agencies, which nonetheless undergirds and perpetuates exploitation. Similarly, Anne Gilliland’s examination of the recordkeeping regimes produced by borderization and Jarrett Drake’s analysis of New Orleans police department documentation in the aftermath of Hurricane Katrina demonstrate how the production of proof is a highly political and deeply contingent process (Gilliland 2017; Drake 2014).

Within the context of Indigenous human rights, archival scholars have also engaged extensively with the relationship between recordkeeping and welfare systems, with a particular attention to the legacies of colonial violence perpetuated through, and sometimes documented in, records of State-provisioned care (McKemmish et al. 2011). As Golding et al. write “government recordkeeping was central to the implementation of the oppressive laws, practices and policies that denied Indigenous Australians their countries, their identities, cultural heritage and languages, suppressing the practice of culture, and breaking transmission lines” (2021a, p 1633). In response to calls to address legacies of violence, archival scholars have proposed multiple responses including establishing a rights-based framework for Care-leavers and Care-experienced people (Golding et al. 2021a, b; Hoyle et al. 2018), radically expanding and pluralizing access to records (Evans et al. 2020; Tropea and Ward 2021), emphasizing the importance of records for Care-leavers’ memory and identity needs (Hoyle et al. 2020), and building more participatory recordkeeping models for care settings (Shepherd et al. 2020). While in a different context of care, our research on recordkeeping in homeless services is informed by this body of the literature on child welfare systems. In particular, this literature points us towards the violence of opacity and blocked access in State care records, and the importance of foregrounding the humanity and rights of those who come into contact with care infrastructures.

### Recordkeeping in homeless services

Homelessness represents a significant, persistent, and growing social crisis in the USA. The total number of unhoused persons rose for the fourth consecutive year in 2020, and preliminary research suggests that the Coronavirus pandemic and accompanying economic downturn will further exacerbate this trend (Joint Center for Housing Studies of Harvard University 2020; U.S. Housing and Urban Development 2020). The formal, national system for addressing homelessness in the USA was created in 1987 with the passage of the Stewart B. McKinney Homelessness Assistance Act (US. PL100-77, 1987). The passage of the law created relatively few new institutions. Rather, existing institutions (nonprofits, state and city governments, and hospitals) and spatial configurations (shelter buildings, churches, and existing housing) were united under a formal, increasingly centralized system.

Since the Homeless Emergency Assistance and Rapid Transition to Housing (HEARTH) Act of 2009, the policy response to homelessness in the USA has been increasingly centralized and data-driven (US. PL111-22, 2009, p 1663). The Federal government has sought to unite the disparate organizations who provide shelter and housing resources to the unhoused into cohesive, geographically bounded Communities of Care (CoCs) through mandating a single set of data-gathering and reporting elements, and by proposing unified goals and strategic aims (Balagot et al. 2019). This unified approach includes a focus on systematic assessment and resource allocation known as the Coordinated Entry System (CES) model, in which housing resources are distributed in order of assessed vulnerability rather than on a “first come, first serve” basis (United States Interagency Council on Homelessness 2015). Together, these two laws (McKinney-Vento and the HEARTH act) created the homeless services system as it exists today—a loosely centralized assemblage of actors connected by funding stipulations, reporting mandates, and recordkeeping technologies. For a more detailed history of the U.S. policy response to homelessness and the birth of the formal system, see Beck and Twiss (2019) *The Homelessness Industry: a Critique of US Social Policy*.

Recordkeeping abounds within homeless services. Virtually every interaction between a social service worker and an unhoused person generates records. Meetings must be documented with case notes, forms and attestations must be filled out to prove disability status or chronic homelessness, benefits must be applied for, and decisions must be appealed. The recordkeeping ecosystem of homeless services blends documentary formats and processes from public housing bureaucracies (income verification forms) with those from medical contexts (psychiatric evaluations). Records in homeless services fulfill both of Matthew Hull’s “broad capacities of documents,'' working both to facilitate administrative control (see Willse 2015) and to construct “subjects, objects, and socialites” (Hull 2012, 256; see Lyon-Callo 2004). Recordkeeping requirements are specified by both federal policy, largely dictated by Housing and Urban Development (HUD), and by State and municipal policies, creating a dense and sometimes contradictory set of demands on social service workers and administrators.

Recordkeeping work in this arena is typified by the case note—a type of record that social service workers produce detailing their interactions with individual unhoused people. These records might include the date, time, length, and setting of the interaction; what the goals of the interaction were and how these related to more long-term projects; what was accomplished; and what next steps are to be taken. The case note, although required to follow various conventions for professional writing, is written free-form into an open text field or even a word processing document. Case notes are not unique to the context of homeless services and exist in a variety of social services and medical contexts.

Another type of record fundamental to our analysis is the “Vulnerability Index—Service Prioritization Decision Assistance Tool (VI-SPDAT)” assessment tool. In the wake of the Great Recession, the recordkeeping apparatus of homeless services changed significantly when HUD introduced a new paradigm for the system called Coordinated Entry. Coordinated Entry asked localities in the USA to adopt, design, and implement centralized systems for resource allocation based on systematic assessment of vulnerability rather than on a “first come, first serve” basis. HUD mandated that each locality utilize an assessment tool to determine which unhoused people were the most vulnerable and should therefore be prioritized for housing assistance and other services; however, HUD did not specify a particular tool or develop its own. While various tools have been adopted, the most common is the VI-SPDAT, an assessment tool, developed in 2013 by the nonprofit consultancy OrgCode and implemented widely in various iterations. The VI-SPDAT and the host of other documentation requirements and forms that are stipulated and generated by Coordinated Entry, are all examples of recordkeeping—a particular set of technological practices crucial to the function of any bureaucratic system.

This research proceeds from the standpoint that the VI-SPDAT and other types of record production and preservation activities in homeless services are intimately linked, forming a recordkeeping regime. This recordkeeping regime is a fundamental part of the homeless services system and can even be thought of as the key unifying component of an otherwise ad hoc assemblage of governmental, NGO, and for profit institutions. In this way, the records and recordkeeping practices of homeless services systems can be usefully thought of as the unifying element of an infrastructure.

## Conceptual framework

### Infrastructure

Infrastructures are, as Paul Edwards writes, “large, force-amplifying systems that connect people and institutions across large scales of space and time” or as Brian Larkin puts it, “built networks that facilitate the flow of goods, people, or ideas and allow for their exchange over space” (Edwards 2002, p 186; Larkin 2013, p 328). Star and Ruhleder pose a classic list of characteristics: Infrastructures are embedded in other social or technical arrangements, reusable, have reach beyond a single event or site, shape (and are shaped by) conventions of practice which are learned as part of group membership, embody technical standards, such as plumbing or electrical codes, are built on an existing base, and become visible when they breakdown (Star and Ruhleder 1996, p 113). Beyond these dimensions, infrastructures are “inevitably imbued with biased struggles for social, economic, ecological and political power to benefit from connecting with (more or less) distant times and places” (Graham and Marvin 2001, p 11). This is to say that infrastructures can cause harm not just during breakdown, but also during business as usual.

As Susan Leigh Starr notes, an infrastructure is a “system of substrates” which becomes more complicated when one examines “the situations of those who are not served by a particular infrastructure” (1999, p 380). According to John Durham Peters, an infrastructural approach is ideal for studying “the basic, the boring, the mundane, and all the mischievous work done behind the scenes” (2015, p 33). What could be a more suitable approach for studying forms in a system that is chiefly characterized by waiting?

The dynamics of infrastructural visibility (when breakdown brought the substrate to the foreground or when breakdown was itself mundane) are of particular importance for our study. Infrastructures vary in their visibility depending on who is looking. While some scholars have focused on how infrastructure disappears for its users, others have highlighted instances of infrastructural hypervisibility. The building that an unhoused person recognizes as an after-hours shelter—a potentially lifesaving respite against extreme heat or cold—disappears into the urban fabric for a person unfamiliar with the infrastructure. Identifying *when* the software, databases, forms, and algorithms of homeless services were visible to the social service workers we interviewed, and when they faded into the background, proved vital for understanding homeless service systems as sociotechnical constellations.

In engaging the infrastructure concept, we join several scholars who have utilized infrastructure as a framework for understanding various aspects of archives, recordkeeping, and data structures. Most of this work examines archives as part of information or knowledge infrastructures, defined variously as “robust networks of people, artifacts, and institutions that generate, share, and maintain specific knowledge about the human and natural worlds” or “​​the network of institutions, people, buildings, and information resources which enable us to turn observation and contemplation of the world into a standardized set of knowledge objects: journal articles and monographs” (Edwards 2010, p 17; Bowker 2016, p 391). This work spans research from the archival science and data curation communities, including work on backlog (Trace 2022), taxonomic practices (Thomer, Twidale, and Yoder 2018), scientific collaboration (Karasti et al. 2010; Korn et al. 2017), and social services databases (Bopp, Benjamin, and Voida 2019), among many other applications. Other work has connected infrastructure to more theoretical debates within archival science, such as Amelia Acker’s (2015) formulation of “biorecords.”

Departing somewhat from the knowledge or information infrastructural approach, we retain an attunement to materiality, the “material tendencies and properties of technological forms and their cybernetic systems” (Rossiter 2021, p 134). That is to say, our research examines both digital PDFs, databases, and communications networks, and physical buildings, paper forms, and practices of transporting and housing bodies. We consider both the intangible and tangible as part of the infrastructure and part of the object of study.

### Homeless services: an infrastructure of last resort

In our work, we use infrastructure as a conceptual framework to analyze the homeless services system as conceptually unified, despite its myriad array of component institutions, technologies, actors, and relations. This infrastructure includes shelters, transitional housing, rapid-rehousing, and permanent supportive housing, wraparound case management, progressive trauma-informed social workers, reactionary Reaganite social workers, backdoor access to section-8 vouchers, warming centers, church floors, public restrooms, and warm- and cool-handoffs to/from endless linkages: veterans Affairs hospitals, domestic violence shelters, community health clinics, medicaid scams, community legal services, the prison system, the emergency room, the behavioral health system, day programs, night programs, inpatient, outpatient, modified intensive outpatient programs, methadone and suboxone clinics, job training programs, gig-economy jobs, and every type of cheap housing, public or private, legal or illegal, that can be imagined. Undergirding and binding together these disparate components is a sea of records: federal and state ID documents, citizenship documents, housing applications, Homeless Management Information System documentation, incident reports, Housing and Urban Development records, Social Security Administration records including social security insurance/disability insurance materials, VI-SPDAT assessments, incarceration records and court filings, and medical records.

Given the myriad array of actors and technologies that makeup the homeless services system, we also use infrastructure as a conceptual framework to examine how sociotechnical systems build on the sediment of previous configurations. As Peters (2015) writes, “infrastructures tend to change incrementally, and have the inertia of previous innovations to build upon. They are improved upon modularly, and clearly illustrate the principle of path-dependence” (p 33). The homeless services system is in some ways very new—it did not exist as such prior to 1987. Even with the passage of the McKinney-Vento Act in 1987, few new institutions were created. Rather, existing institutions, actors, and spatial configurations were drawn together in a formal, increasingly centralized system. The “inertia of previous innovations” is everywhere in homeless services, and the system is always an amalgam (Peters 2015, p 33).

Finally, an infrastructuralist approach is also useful for our study because it helps account for the actions and agencies of nonhuman actors in the homeless services system. As Lisa Parks and Nicole Starosielski write in *Signal Traffic*, “humans are but one part of broader infrastructural formations” (2015, p 10). In the USA, non-human actors often function as the glue that holds the people and institutions of homeless services together; these non-human actors include a federal funding apparatus and associated recordkeeping and auditing practices that structure and formalize aspects of the infrastructure. This theoretical nuance helps to situate the tangled functioning of records in the homeless services system where, as we will later show, human workers increasingly struggle to exert control.

## Methods

### Methodological orientation

To understand how records function in the homeless services system, this research utilizes a multimodal approach, combining interviews and artifact walkthroughs. Following what Kalindi Vora calls a “juxtapositional reading practice,” this research combines social service workers' experiential knowledge gathered from interviews with collaborative close reading of specific forms via artifact walkthroughs, aiming to produce “histories of the present” (2015, p 18). This methodological orientation views and records both as embedded in work processes and as media objects in their own right. This multimodal approach fits well with the theoretical frameworks provided by the archival literature on recordkeeping, and the media studies and information literature on infrastructure, as scholars working from both standpoints, have utilized a variety of discursive, ethnographic, and interview-based methods.

The research design was partially informed by the first author’s prior experience as a social worker in homeless services. This prior experience extended the first author some of the benefits and challenges of an insider-researcher position. For instance, prior experience facilitated building trust with participants and provided a basic level of familiarity with some of the recordkeeping formats and technologies used by participants. It also potentially introduced additional blindspots for the first author, which they sought to mitigate through frequent consultation with the second and third authors, who did not have prior insider experience. Some aspects of insider-researcher status were also not applicable to the first author, for instance they do not have role duality (Unluer 2012) and were also unfamiliar with many of the specific tools and technologies that participants described.

### Ethical considerations

Designing a study to tackle our research questions posed a number of ethical challenges. Many of the social workers who participated in our study experience various forms of social and economic precarity, including health risks associated with being essential workers during the Covid-19 pandemic. Additionally, participants faced significant potential harms, particularly reputational harms, from speaking honestly about their professional lives.

In order to mitigate these risks, we developed an informed consent process in which the first author discussed potential harms with participants, highlighted the option to speak off the record, and solicited feedback in determining what might be potentially identifying for purposes of anonymization. Interviews were conducted remotely via video-call, were transcribed and anonymized, and all research data was stored securely. Participant details were stored separately from de-identified transcripts, and original audio files were deleted once transcription had been satisfactorily completed. This study was reviewed by the University of Michigan human subjects research unit and approved as an exempt study.

The voices of people with direct, lived experience of homelessness are largely missing from this paper (a few of our participants had previously experienced homelessness). Data collection for this study was completed between July 2021 and September 2021 during the Covid-19 pandemic (coinciding with the Delta-variant wave in the USA). Despite wanting to also interview people currently experiencing homelessness, we determined that it was unreasonably risky to do so in-person and logistically impractical to do so via video or phone calls. The absence of these voices is a significant limitation to this study, which we hope to rectify through future work. We address these limitations in the discussion section.

### Participant recruitment

We recruited interview participants by email and phone through professional networks in the homeless services field. Next, we identified additional participants through snowball sampling, following connections and recommendations from early participants in an effort to include specific perspectives that appeared to be missing “as concepts emerge[d],” based on theoretical sampling (Bernard 2011, p 435). All study participants have direct experience working in the homeless services field in roles including but not limited to case management, housing administration, HMIS administration, program coordination, homeless outreach, social worker, and city government officials. Recruitment included participants representing non-profit and state institutions with a variety of years of experience in the field. To ensure a diverse group of participants across lines of race, gender, and age, participant selection followed a firmly interpretative method and did not aim for any sort of random sample. Rather, through speaking with people with extensive experience with the documentary forms identified during the conceptualization of this project, this study sought to better understand those recordkeeping practices and learn of additional ones. The following table lists our participants, identified by pseudonyms, along with their years of experience and broad role within the system (Table 1).

**Table 1:** Participant details

P#	Participant	Years of experience	Broad role
P1	Olivia	0–5 Years	Large nonprofit
P2	Elizabeth	5–10 Years	Small nonprofit
P3	Emma	0–5 Years	Large nonprofit
P4	John	15 + Years	City Government
P5	Sophia	10–15 Years	Large nonprofit
P6	Isabella	15 + Years	Small nonprofit
P7	Grace	5–10 Years	Large nonprofit
P8	Michelle	0–5 Years	Hospital
P9	Amanda	5–10 Years	Large nonprofit
P10	Michael	10–15 Years	Large nonprofit
P11	Chris	5–10 Years	Large nonprofit
P12	Stephanie	5–10 Years	Small nonprofit
P13	Lisa	5–10 Years	Large nonprofit
P14	Amber	0–5 Years	Large nonprofit
P15	Jamie	5–10 Years	Large nonprofit

### Data collection

This study generated its insights from fifteen semi-structured, one-hour remote interviews via video calls. Participants gave consent to record the interviews. We anonymized all participant identifying information through the use of pseudonyms for participants, organizations, and employers. We utilized a semi-structured interview protocol (Appendix 1) to direct the conversations and approached participants as knowledgeable collaborators with valuable experiences. For those who were interested, we retained contact information so that we can provide them access to the results of the research.

Each interview also included an artifact walkthrough, asking the participant to bring a form or tool from their recordkeeping work “to recreate a specific process… using artifacts from the actual process to stimulate their recollection” (Raven and Flanders 1996, p 4). The goals of the artifact walkthrough were twofold: to gain additional insight into the processes of recordkeeping in homeless services systems and to provide a prompt for discussion and collaborative thinking between participant and researcher.

This multi-modal combination of interviewing and artifact walkthroughs is in keeping with similar investigations of infrastructures in that it examines both the people and the artifacts that make up the system and allows for thinking about the relationships between recordkeeping, infrastructure, and human actors across different scales and sites.

### Data analysis

We analyzed the interview transcripts utilizing inductive thematic coding, borrowing loosely from Adele Clark’s (2005) approach to situational analysis, and drawing inspiration from works like Nicole Starosielski’s *The Undersea Network* in which interview data makes up part of a “multivalent model for studying distribution systems,” resulting in deepened insight (2015, p 22). We then analyzed the coded data alongside the forms and tools identified through artifact walkthroughs, iteratively memoing and reworking our themes until they fit the data well and additional refinement did not facilitate further insight (Braun and Clarke 2006, p 92). This approach aimed to not only gain a greater understanding from interviewees, but also remain committed to “taking the nonhuman—including discourses—in the situation of inquiry seriously” (Clarke 2005, p xxxvi) We took this approach in part as a recognition of the limitations of interviewing for achieving the deep comprehension of records that this research aspires to, given that conceptualizing these practices in this way was counterintuitive for some participants. While this issue was partially mitigated through careful theoretical sampling and utilizing a well-designed interview guide, a deeper level of understanding was obtained through combining interview data with collaborative close reading of forms and recordkeeping tools.

## Findings

Our findings show how recordkeeping functions in the ‘infrastructure of last resort’ from the perspective of social service professionals who work within it. Through their experiences, we offer an account of how records function as logistical tools for increasing efficiency within the homeless services system and the impact this has on those who provide care to unhoused people. The findings proceed as three arguments. First, we examine how recordkeeping functions as an accountability measure that makes care work legible to supervisors and funders. Second, we contend that a substantial portion of recordkeeping fulfills a second, less obvious function—logistics. Finally, we trace a set of practices that our interlocutors deployed in the face of the demands of logistics, practices we frame as translation work.

### Records for accountability

Records abound in homeless services work practices. Social service workers are constantly preserving traces of their activity and interactions, jotting down notes, and completing formal and informal documentation. In the course of a single, one-hour meeting with an unhoused person, a case manager might create a profile and enrollment in a Homeless Management Information System (HMIS) database, complete half a dozen paper intake and consent forms, narrate the person’s housing history on a Proof of Chronic Homelessness worksheet, send email, paper, or web-form based referrals to legal or medical clinics and then, document the whole meeting in shorthand as a case note. This saturation of the working day with records is not limited to first meetings. Subsequent interactions generate their own combinations of digital and analog records, as social service workers and their unhoused clients complete housing applications and vulnerability and psycho-social risk assessments, initiate algorithmic matching processes, and negotiate a vast web of services and technologies in order to maximize the unhoused person’s chances of receiving aid in chronically under-resourced systems.

Many of these recordkeeping practices are, at least at face value, intended to produce a multiplicity of accountabilities by making care work legible both to supervisors and to external institutions providing funding and oversight. As Michelle, who worked with unhoused and formerly unhoused clients in a case management role put it:

Everything is about documentation in this job, so if I wasn't to write a note, then it's almost like it didn't happen, because then it doesn't get billed and then there's nothing backing that it was a service that was provided.

For social service workers, recording their interactions with clients is important in different ways for different audiences. For supervisors and management, records proved that their employees were meeting regularly with the clients assigned to them and fulfilling their responsibilities. For funding bodies, including Federal and State sources, as well as nonprofits and philanthropic organizations, records served as evidence that goals were being met and grant funds spent properly. Similarly, for development officers at small non-profits within the homeless services system, recordkeeping created data points that they leveraged in grant-writing and donor relations work. For city-level administrators, recordkeeping at the organizational and individual worker level enabled some oversight and evaluation, particularly quantitative evaluation and resource allocation.

Records also helped provide accountabilities within workplaces and teams. Case notes, for example, enabled continuity in client care. Good notes helped social service workers track client goals and progress over time, while helping coordinate handoffs between social service workers within a single organization during typically high rates of staff turnover. Sophia, who worked at a large men’s shelter and had over a decade of experience in homeless services, articulated that:

Notes are useful... it makes sense intra-agency to have that information so that if I'm working with someone and I'm out, my colleague can go in, look at my notes, see what's going on, and show up for that person appropriately.

The usefulness of case notes here produces a further incentive for social service workers to document their interactions, as a way of helping their clients *and* their colleagues succeed. This range of motivations and responsibilities reflect Yakel’s (2001) observation that recordkeeping practices often produce “multiple accountabilities” rather than only unidirectional expressions of power and responsibility (p 33). The production of accountability is the stated aim of a significant amount of the recordkeeping work within homeless services.

### Records for logistics

However, as archival scholars have long argued, records have functions and impacts far beyond, and sometimes contradictory to, their official purposes. Participants attested that recordkeeping often was effectively unrelated to accountability, instead serving the logistical purpose of helping move unhoused people between sites in the infrastructure. This second function involves the production and circulation of records for the purposes of facilitating and tracking the movement of unhoused people through the homeless services system.

To lay out this argument, let us quickly describe the path an unhoused person might take through the infrastructure of last resort. In order to access services, a person typically must report to a central intake center where a social service worker asks them to sign a consent form and then, enters them into an HMIS database. Within that database, the worker then creates a referral to an emergency shelter, provides the unhoused person with directions to that shelter, and sends them on their way. Without a referral, the person will not be able to access the vast majority of shelters. Even with a referral, the person can only access that specific shelter—even if it is in an unfamiliar part of the city or inaccessible via public transit.

Shortly, after arrival at a shelter, an unhoused person will meet with a case manager, housing specialist, or other social service worker who will complete a housing assessment with them. This series of digital and paper forms acts as a “low-tech algorithm,” sorting unhoused people into tiers of eligibility based on length and frequency of homelessness, age, disability, gender, sexual orientation, HIV-status, and a quantitative vulnerability score. If they are determined to be eligible for housing assistance, such as a rental subsidy, they then wait in shelter until they are matched with an eligible vacancy (this might take anywhere from a few months to a few years). Then, a social service worker completes a lengthy packet of application materials with them and forwards these to the housing service provider for processing. If accepted (and it is a big “if”), the unhoused person can move from the shelter to their housing. As with the shelter referral process, recordkeeping is a prerequisite to moving out of shelter; an unhoused person cannot move into housing with which they are matched until their packet of forms and documentation is accepted.

The visible purpose of these recordkeeping processes is accountability. For example, a chronic homelessness form (completed during the housing assessment) is intended to make non-profit housing providers accountable to their funding bodies and make unhoused people accountable to housing providers (and the broader public) as a form of means testing. Our participants’ experiences, however, indicated that accountability is not the primary function of these records. Rather, these records function to facilitate and delineate the movement of unhoused peoples’ bodies through the system.

One of our participant’s experiences with housing packets illustrates this well. Amanda, a social worker who had helped implement coordinated entry at a large non-profit housing provider, described how success in helping clients obtain housing hinged on staff being, “well-versed in what was needed, what documentation was needed, as a full packet.”

Creating this packet would include making copies of an unhoused person’s “ID, social security card, and birth certificate,” (or helping them obtain these documents in the first place) as well as “getting connected to a mental health provider or someone that can complete [proof of disability] documentation and getting all the chronic homelessness documentation together, [which] often was a really clunky part.”

Gathering and collating these records quickly is important because, for the housing provider, the process is depersonalized and must happen according to a strict timeline. As Amanda described, housing providers:

get a referral, a match, and everything is de-identified. Referrals just show client IDs and they can't see somebody's name or where they're staying. They get a match within two days from [the city automated matching software] that says, ‘hey, here's this person, here's who to contact, you have two days to do it.’ They have five days to respond. And then it's structured so that if things aren't happening it gets closed out and onto the next one… because unfortunately [the provider] would have to return those matches if the support couldn't get the stuff in on time.

Once a match is ‘closed out,’ the unhoused client will have to wait until they are algorithmically matched to another unit. While unhoused people technically have the right to be matched as many times as necessary, the intense shortage of housing resources and the opacity of the matching process mean that they may not be matched again for many months and that the length of that wait is a mystery. This account of the timeline and flow of records evinces how the logic of logistics has displaced that of accountability in homeless services. The pressure to quickly distill the complexities of homelessness into a series of stubbornly resistant formats produces an environment in which efficiency is valued more than an accurate accounting of individual situations.

While accountability is still part of the function of records in this infrastructure, it is not the primary function. Rather, to borrow a phrase from media scholar Cornelia Vismann, these records “transfer,” working logistically to “sort and engender circulation” of human bodies (2008, p 6). Housing application packets function like shipping mandates—accompanying the unhoused person’s body through space and dictating which spaces they can occupy and under which conditions. HMIS databases have much in common with inventory and supply chain management software, coordinating the arrival and departure of unhoused people and managing the availability of beds and funding streams (Rossiter 2017). Following John Durham Peters articulation of “logistical media,” we might call records in this mode *logistical records*. These records work, as Peters writes, “to organize and orient, to arrange people and property, often into grids. They both coordinate and subordinate, arranging relationships among people and things” (2015, p 37).

The appearance and persistence of this second, less visible function of records is not unique to homeless services but appears in other infrastructures as well. Jesse LeCavalier has called this phenomenon “logistification,” a process which aims to increase speed and efficiency in an existing infrastructure by working “to flatten, connect, smooth, and lubricate as it organizes material in both space and time” (2016, p 6). This process involves attempting to increase the rate of flow within an infrastructure without fundamentally changing the infrastructure itself, or as LeCavalier writes, “to exceed the limits of infrastructural systems while simultaneously relying on them (and being defined by them)” (2016, p 50).

In the context of homeless services, “flow” describes the circulation of unhoused persons through the system. Conceptualizing homelessness as a problem that can best be solved by increasing the rate of flow represents a logistical dream, where seemingly intractable social problems are bypassed, at least partially. In this logistical vision of homeless services, recordkeeping plays a crucial role in producing unhoused people as neatly packaged individual ‘units’ which can be moved through the system as interchangeable modules. Logistical recordkeeping functions to flatten the complex lives of unhoused persons in ways that allow for the smooth and efficient circulation of human lives both in information systems and in the prescribed urban spaces allocated to unhoused people.

### Breakdown

Logistification significantly improved aspects of the homeless services system when it worked well, particularly through centralizing disparate resources across organizations. However, the logistical dream did not always function as promised. A number of participants expressed anger and frustration at the limits that logistical records imposed. One was Emma, who had worked at a men’s shelter for several years and had extensive experience with housing allocation. Her description of the paperwork process for moving someone into housing after an algorithmic match exemplifies some of these frustrations:

I have a client who's been in the shelter since last July and has been matched… to housing at [subsidized apartment complex run by large non-profit] since February, and they started telling us we need to send back different versions of the paperwork.

We sent them three different versions of the paperwork, and then there was silence for like a month and a half while they had some random staff turnover and they did not communicate about what was happening. And then they were like, ‘Oh, your application is good… but now this person's award letter is out of date, so we need a new award letter.’ So we had to get him a new [Social Security Administration] award letter.

We got it, we sent it to them, they were like, ‘Oh, now these dates from this application that we sent in are out of date, so you need to send it again,’ which we did, which was at the beginning of June, and he's still in the shelter during a pandemic, and disabled, and has schizophrenia.

Emma’s struggle trying to obtain housing for her highly vulnerable client illustrates a breakdown in the infrastructure and the impact of logistical failure. Despite meeting all eligibility requirements and having successfully navigated the housing match process, her client is unable to physically move into safe, dignified housing because of delays in processing logistical records. Because the movement of unhoused people is linked so tightly to the circulation of logistical records, a medically vulnerable unhoused person with a serious mental health condition remained in a crowded shelter during Covid for more than five months *after* being approved to move to a safer, more dignified space.

Subsequent conversations with other social service workers indicated that breakdowns were common, rather than a rare exception to the rule. Breakdowns manifested as delays and communication failures, but also in situations where the complex realities of homelessness proved difficult or impossible to articulate given the formal constraints of logistical records. In these cases of breakdown, our participants often reported an iterative negotiation where they worked to fit the life experiences of an unhoused person into the categories and flows of the infrastructure of homeless services. This translation work was a vital yet largely unrecognized aspect of their labor, as they constantly staved off infrastructural breakdown and its consequences.

### Translation work

The social service workers interviewed for this study clearly recognized the vital importance of recordkeeping work—how even seemingly innocuous discrepancies like a missing middle initial could result in an unhoused client spending additional months or even years without permanent housing. As recordkeeping work became increasingly vital to helping their clients achieve successful outcomes, they developed a heterogeneous set of strategies to navigate this new work, strategies which we call acts of translation.

Taken as a whole, these acts of translation represent ways that our participants were able to maintain their commitment to advocacy and to express their expertise within the confines of recordkeeping forms and technologies. These strategies came in various forms. Common across them was that the social service worker was doing some sort of translation work—either working to make the infrastructure legible to the client, the client legible to the infrastructure, or both. This translation work is essential, as Elizabeth the social services manager explained, because:

The nature of people living in crisis situations [is that] these might be people that have master's degrees, but you throw them in a situation where they desperately need this resource and it's really hard to read all of the words… So having people that are able to translate these documents is really helpful for this population, for anyone really in crisis.

These acts of translation are aimed at mitigating a variety of issues imposed by the rigid format of the recordkeeping tools common in the homeless services infrastructure. The rigidity of formats in the system is typified in many ways by the vulnerability assessment tool used in the city we studied, the VI-SPDAT. A social service worker administers the assessment—a five page form with twenty-seven yes/no and “how many times” questions—to an unhoused person by asking the questions verbatim and recording the answers in a locality’s HMIS database. The unhoused person receives a score which determines their tier. *Tier one* means no eligibility for housing assistance, *tier two* means eligibility for temporary assistance, and *tier three* means eligibility for long-term assistance. Unhoused people who score in tiers two and three are entered into an algorithmic matching system which pairs unhoused people with available units, factoring in their level of vulnerability, disability status, and other information. The primary factor in matching is vulnerability score, which is the basis for prioritization. Social service workers are prohibited from deviating from the assessment script and are forbidden from disclosing the tiered system or any other details of the housing match process.

For an unhoused person, the vulnerability assessment process can be both traumatic and bewildering. Emma, the men’s shelter worker, called the assessment interview:

A kind of a miserable process because you're asking people to talk through all of their traumas and vulnerabilities. I've definitely seen it make people cry. I've seen it make people angry. And then the most common reaction I see is that it makes people really shut down.

In addition to being emotionally intense, the assessment interview is also confusing. Emma described how the assessment encouraged a counterintuitive disclosure of “barriers”—aspects of an unhoused person’s lived experience like substance abuse or involvement in the criminal justice system that are categorized as obstacles to housing:

I let people know ahead of time that I have to read the questions exactly as they're written, but they should feel free to ask for clarification. I'll say, "it's definitely in your interest to be as honest as possible." Because I think that it's really confusing for people to know when, in a system, talking about a barrier is going to present a barrier, and when talking about a barrier is going to present an advantage.

Emma’s subtle skirting of the assessment interview protocol, through encouraging her clients “to be as honest as possible,” is an example of an act of translation. Her translation work mediates between the infrastructure and the unhoused person. While at many sites in the infrastructure disclosing ongoing substance use could cause an unhoused person to be denied services, in the context of the VI-SPDAT, disclosure will improve the client’s vulnerability score. Even though Emma does not have a way to directly input professional opinion into the process, she is able to influence the outcome through this translation work. Other participants reported similar strategies. Stephanie’s translation was also focused on mixed signals, as well as on trying to overcome what she saw as poorly worded assessment questions:

The hard part is that they want you to use the language that's in there. And the language is meant to be colloquial and casual, but it also ends up being confusing. The other part is when you first meet [an unhoused client], a parent especially. You ask a question that might garner them a point, and they don't know what the point system is, they just know you're asking these questions. The question sounds like they’re not being a great parent, and so they’re not inclined to answer in the way that they might [score a point.]

Similarly to Emma, Stephanie was able to influence outcomes through framing the function of the assessment questions, even though she could not directly impact the assessment. She saw this translation work as being somewhat successful, to the extent that:

Over time we just got really adept at the VI-SPDAT, and we were able to explain the questions a little bit better and explain to people the purpose of it and go a little less by the script.

Amber, a case manager at a large non-profit who worked with unhoused women, also began changing her reading of the form, abandoning a verbatim reading:

When I ask the questions for it, you're supposed to ask them exactly how they're phrased. I will typically ask it in a way that pertains more to the individual in front of me, sort of knowing what their answer should be for that question. Because some of [the questions] are just phrased so oddly that they're going to score a lower score when they sort of deserve to be higher.

Other acts of translation were less focused on the immediate recordkeeping technologies themselves and were more aimed at helping clients understand the infrastructure more clearly. As Stephanie noted:

But with the VI-SPDAT… the hard part is you can't really overtly tell somebody like, ‘Hey, I need you to tell me that thing that you told me the other day, but in this context so that I can [get you a higher score].’ It's like a little bit of a mystery and so people are kind of left being like, ‘I don't know. I took that thing the first couple of days I got here and now I don't know why I'm not being picked up.’”

Jamie, a housing administrator at a large non-profit, described a similar process of trying to explain the counterintuitive reality that, although a failed drug test technically disqualified a client from housing, in practice that was not the case. She stated,

We do drug testing for our sites. But for everything that could cause a rejection, clients are able to appeal that rejection. So for example, if we have someone who gives a positive drug test, they can come back to us with an appeal letter saying, "Hey, maybe I had this much time sober ahead of time and then had a relapse. Here are my supports. Here's what I'm doing moving forward." And that's fine.

This translation work, whether Elizabeth’s translation of welfare application materials, Emma’s subtle bending of VI-SPDAT assessment rules, or Jamie’s explanation that rejections are not really rejections, is prevalent in homeless services, and points towards the widespread failures of logistification. For every story of efficiency in our participants’ narratives, there was a story of breakdown, workarounds, and impossible expectations.

Translation work is a testament to the ambivalent position of many social service workers in the infrastructure of last resort. Social service workers’ professional lives are increasingly filled with data entry and recordkeeping work which facilitates the logistification of the broader homeless services system. This increase in recordkeeping work diminishes the time they can spend directly interacting with unhoused clients as full people, rather than as representational abstractions via forms and assessments. Translation work was an avenue for some workers to inject their expertise and creativity into the work process through mediating between the complex lives of their unhoused clients and a rigid, partially automated recordkeeping system. However, many of our participants reported little satisfaction from this mediating work, even when it was successful—they mostly felt frustrated at having to come up with these strategies in the first place.

## Discussion

To examine recordkeeping within the homeless services system, we moved beyond analyzing records through an accountability lens to consider how records *function* within a larger infrastructure, sometimes in ways that contradict their official purposes. By focusing on the infrastructural functions of records, our analysis of the social service workers’ interviews found that while recordkeeping sometimes did promote accountability (between case workers and their supervisors for example); a more significant function of recordkeeping was facilitating the efficient movement of unhoused people’s bodies through space. We argue that the prevalence of recordkeeping as a tool for efficiency serves as evidence of logistification—a process of expediting flows through an existing infrastructure with the goal of increasing speed and efficiency.

Although many of the social workers interviewed saw aspects of logistification (like centralization of housing resources) as positives, their experiences also reflected acute failures of the infrastructure and long-term erosion of their ability to provide care. Our participants, particularly those whose work involved direct and daily interaction with clients, expressed how important it was for them to be invested in the care of others. Thus, they reacted to logistification by using informal work practices that allowed them to advocate for their unhoused clients in new, technologically mediated ways.

These recordkeeping practices function as acts of translation between the categories and ways of knowing imposed by the system and the lived experiences of unhoused people. Social service workers saw translation as a form of care and important for affirming their unhoused clients' humanity in the face of a dehumanizing bureaucracy, while also providing avenues for discretion in an increasingly centralized system. These acts of translation were vital not only because they enabled the social service workers to more fully care for their clients under the conditions of logistification, but also because it allowed them to reclaim a sense of expertise and control.

Through our participants’ experiences, we came to see care as an ambivalent concept, in the sense that it was truly multi-valent. Similar to the concept of radical empathy (Caswell and Cifor 2016), some participants described trying to preserve universal positive regard for their clients. At the same time, care was not universally positive. Some manifestations of care in their experiences were instead linked to retaining control as their job became increasingly logistified and to propping up dysfunctional aspects of the infrastructure. Thus, they expressed a complicated notion of care that was simultaneously about helping people get their needs met at the most basic level and about the realities of working within, and sometimes against, an infrastructure that limited their control and undervalued their expertise.

Yet, while the social service workers recounted their translation work and use of informal work practices to advocate for their unhoused clients, many of their narratives related to recordkeeping also revealed evidence of a system under strain. As the infrastructure of last resort is pushed to, and beyond, its limits, social service workers experienced this strain as increasingly untenable work conditions. Social service workers largely saw logistification as interfering with their ability to help the unhoused people they work with, while furthering and obscuring the structural violence that unhoused people face.

The experiences shared by the social service workers highlight how these seemingly banal interactions with highly mundane technologies—paper forms, applications, and reports, HMIS databases, excel spreadsheets—have huge impacts on the lives and life chances of social service workers and the unhoused people they serve. Failures in these processes, such as the inability of discharge paperwork to capture the complex needs of a person being expelled from a shelter or a lengthy delay in processing an application for permanent supportive housing, are constant. What might appear to be minor problems in the abstract and can have an enormous impact in the concrete. When a person “is not served” by the infrastructure of last resort, the effects can be devastating.

In 2020 alone, more than 7500 unhoused people died in the USA (Fowle and Gray 2021).

### Study limitations and future work

The absence of direct testimony from people experiencing homelessness and its attendant structural violence is a significant limitation of this research. While social workers have vital and illuminatory perspectives, particularly given their familiarity with the ‘inner workings’ of recordkeeping processes, they experience the impacts of logistification very differently than their clients. To achieve a truly rich understanding of the infrastructure of last resort, analysis of social workers’ perspectives needs to be coupled with empirical research focused on people experiencing homelessness who must navigate the infrastructure to receive potentially life-saving services.

In order to include direct testimony from people experiencing homelessness, our future work will focus on designing a study to address the following question: How does logistification in homeless services systems impact unhoused people, and how is this impact differentiated based on geographic context, familial status, race, gender, age, and sexual orientation? The aim of our future work is to bolster an understanding of how interactions with recordkeeping tools and processes impact unhoused peoples’ struggles to obtain housing, and provide insight into how social service recordkeeping could support more just outcomes. The research that we present here lays a foundation for this future research which will be better placed to propose solutions to the issues identified here, such as policy changes in support of more liberatory archival practice.

## Conclusion

A deep tension exists at the heart of homeless services work. Social service workers we spoke with felt that the immensity of recordkeeping detracted from their ability to provide care for their clients, and a responsibility they saw as the core of their work. At the same time, they also recognized that effective advocacy on behalf of their clients increasingly required strategic recordkeeping practices.

This article explores this tension while engaging and expanding connections between archival science and theories of infrastructure. It adds to a growing body of empirical research within archival studies examining the impacts of recordkeeping in public sector contexts. Through interviews and artifact walkthroughs with social service workers, we illustrate how records and recordkeeping practices function in the context of homeless services, sometimes in deeply counterintuitive ways, and how high the stakes of such recordkeeping practices are.

Analyzing the experiences of our participants through the lens of infrastructure made the homeless services system visible in new ways. Much of the recordkeeping work to which social service workers must devote increasing portions of their working hours is ostensibly oriented towards producing accountability. However, we found that while some of this work does fulfill accountability purposes, much of it is instead related to a different form of administrative control—facilitating the circulation of unhoused clients through the infrastructure.

‘Thinking infrastructurally,’ we identify this process as logistification. Through infrastructure, we keep the broader context of homeless services (its histories, materiality/immateriality, conglomeration of human and non-human players) in the frame, even while focusing on the individual stories and experiential knowledge of our participants. In addition to tracing how recordkeeping practices function, we also considered the ways in which they break down, and how social service workers navigate a logistified infrastructure via tactics, we term translation work.

The ubiquity of this mediating work, between the categories and ways of knowing imposed by the system and the lived experiences of unhoused people, highlights the social complexity of recordkeeping processes and underscores how unhoused people’s life chances can hinge on minute detail. Our findings related to logistification and translation work have important implications for the study and practice of recordkeeping within social service and care work. In the broadest sense, our participants’ narratives offer a compelling case for thinking critically about the function of social service bureaucracies. For any ecology of records, they ask us to consider, does this alleviate harms, or exacerbate them?

## Appendix 1

### Interview protocol

Background:

- What is your background or experience in social services?
- Why did you go into social services?
- How did you begin working in homeless services?
- Could you please tell us a little about your educational background?
- What is your current position/last position held?
- [Follow-up if necessary] Is your employer a non-profit, city/state gov’t, faith-based organization, or someone else?
- What do you see as the core or most important part of your work?

Records:

- Can you walk me through the documentation process you undergo with a typical client? What documents do you need from them, what forms do you need to complete with them, what systems do you need to input information into?
- Could you tell us a little about the types of records that you handle in the course of your day to day work?
- How much of your job do you feel pertains to record keeping?
- How do you feel about record keeping?
- How often do you interact with the personal documents of your clients?
- In what situations are you asking for access to your clients personal documents (such as identity documents, citizenship documents)
- What are your clients general reactions to being asked for their personal records?
- Do your clients ever ask about case notes or case files created on them? Or any other forms or assessments? Ask for copies, ask what is being written in them, etc.?
- Do you have any experience using assessment tools or forms?
- Example: VI-SPDAT, TAY-VI-SPDAT, or PQ9 or similar?
- Could you tell me about a time when a recordkeeping or documentation system is inconvenient or doesn’t work as intended?

Artifact Walkthrough.

- Okay so for the last part of our interview I’d like to do what’s called an artifact walkthrough, where you take me through a blank form or blank document that you use in your work. When we spoke about this previously you said you would like to walk me through [insert form name here]. Is that still alright with you?
- So can you set the stage for me? In what context would you use this form?
- Okay, and so say that I’m the [client/participant/colleague] in this situation. Can you walk me through how you would complete it?

Snowball Sampling:

- I’m interested in speaking with people you know who also have experience in this system. If you think of anyone, would you be willing to share this flyer with my contact information with them?
